# Compound grafting of residual vascularized fibular head flap to reconstruct contralateral lateral malleolus: A case report

**DOI:** 10.1097/MD.0000000000009501

**Published:** 2017-12-29

**Authors:** Peiji Wang, Man-Yi Cui, Jiaju Zhao, Jun Lv

**Affiliations:** a The Second Affiliated Hospital of Soochow University, Department of Hand and Foot Surgery; b Department of Orthopedics, The East District of Suzhou Municipal Hospital, Suzhou, Jiangsu, China.

**Keywords:** complicated lateral malleolus defect, flap, transplantation, vascularized fibular head

## Abstract

**Rationale:**

Serious external ankle injuries caused by high-energy trauma are often associated not only with bone defect but also with a loss of skin and soft tissue that seriously affects ankle function. Therefore, it is particularly important to reconstruct the external ankle.

**Patient concerns:**

The patient was a 64-year-old man with destructive injury of both lower extremities due to a machine accident. His left ankle and heel bone, along with the soft tissue, were torn off, and the peripheral blood supply and sensation to the toes of the left foot were lost concerns.

**Diagnoses:**

Complicated lateral malleolus defect.

**Interventions:**

We used a composite tissueflap taken from thefibular head on the left side to reconstruct the rightfibular lateral malleolus.

**Outcomes:**

With the help of a prosthesis, the patient regained basic walking function by the 1 year follow-up.

**Lessons:**

Using a combined compositeflap of the vascularizedfibular head to reconstruct the lateral malleolus is a good choice.

## 1 Introduction

Serious ankle injury may cause bone defect with soft tissue defect, it is important to improve the quality of life of patients by reconstructing soft tissue defects, as well as the form and function of the ankle joint.^[1]^ To keep the ankle stable and prevent the occurrence of traumatic arthritis,^[2]^ anatomical reduction is demanded as much as possible by surgery. For chronic or infected wound, we should control the infection and cover the wound first,^[3]^ then graft bones in second-stage operation. In that way, adjusting the reduction of fracture may affect flap blood supply. We adopt fibular head composite flap with blood supply to reconstruct the external ankle,^[4,5]^ because they have similar shape and articular surface to form the ankle, the flap can cover the wound at the same time.

## 2 Case report

**Figure 1:**
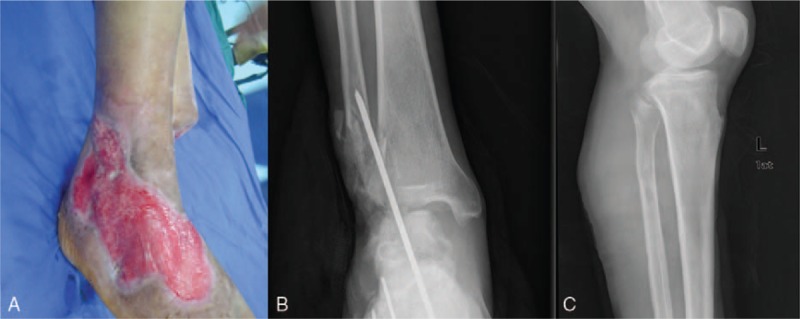
(A) After 2 months of treatment, the wound on right dorsum pedis was healing well with no infection. There was still about 20 × 10 cm skin and soft tissue defect. (B) After previous treatment, other fractures were fixed rigidly, except the external ankle of the right left. (C) Left capitula fibula is complete, and the length is enough.

The patient was a 64-year-old man with destructive injury of both lower extremities due to a machine accident. His left ankle and heel bone, along with the soft tissue, were torn off, and the peripheral blood supply and sensation to the toes of the left foot were lost. We amputated his left leg below the knee. The left fibular head was retained after the left foot operation. Two months later, the patient came to our hospital for additional treatment after debridement of necrotic tissue operations twice. There was still approximately a 20 ×10-cm area on the right foot and right external ankle exposing the distal fibula fractures. X-ray showed that the right external ankle bony defect and the right medial ankle mortise widened; fortunately, the left fibular head was retained after left foot amputation (Fig. 1). We reconstructed the right ankle using vascular anastomosis of the fibular head and a flap taken from the left stump. The Ethics Committee of the Second Affiliated Hospital of Soochow University approved the study.

**Figure 2:**
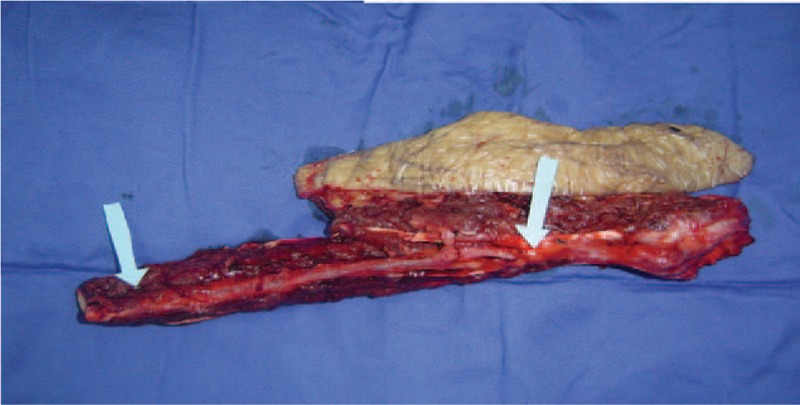
Contralateral vascularized fibular head flap was taken carefully, diameters of blood vessel enable them to suture up.

**Figure 3:**
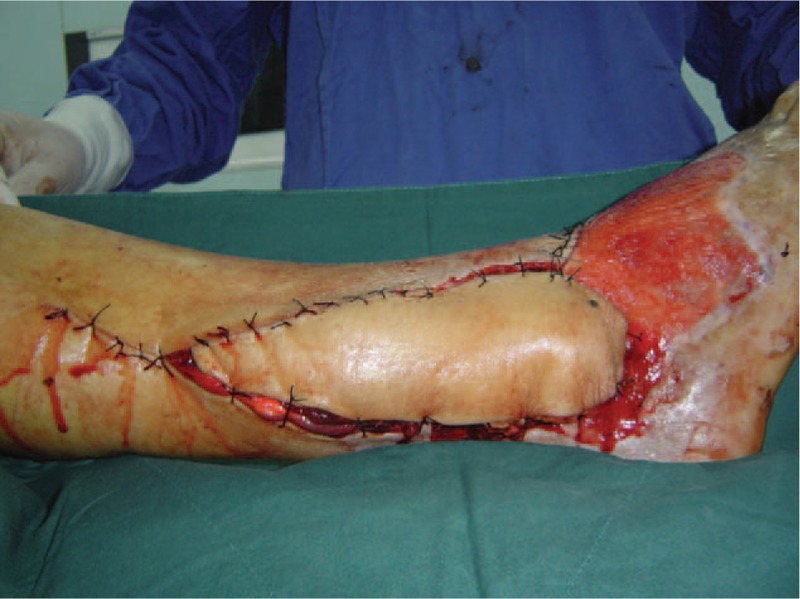
The flap has sufficient bulkiness and a well skin color and texture match of the surrounding tissue.

We used an ultrasonic Doppler instrument (Model: ES-1000SPM Hayashi Denki Co., Ltd, Hong Kong China) to find the perforating branch of the peroneal artery before surgery, and near that location we created a flap approximately 20 × 10 cm in size according to the right foot wound (Fig. 3). Surgery was performed with the patient under general anesthesia. The patient was placed in the supine position on the operating table with the left knee joint slightly bent and the left lower limb stump in internal torsion. The incision began in the popliteal space, swept down to the fibular head, and continued along the lateral peroneus muscles to the stump extremity. We cut the skin and subcutaneous tissue, then separated the peroneus longus and soleus muscles. We found the nervus peroneus communis on the inner posterior margin of the biceps femoris tendon and dissociated it for protection. The peroneal perforator was located in the intermuscular space between the peroneus longus and triceps surae muscles, then we freed the peroneal artery and 2 accompanying veins. We cut off the biceps femoris tendon, fibular collateral ligament and other muscle tissue attached to the fibula, extracted the fibular head, and then divided the superior tibiofibular joint. On release of the tourniquet, bleeding spots on the fibula and residual muscle tissue showed that the blood supply was satisfied. Therefore, we could then cut off the vessels and take the fibular head (Fig. 2). We sewed the biceps femoris tendon and fibular collateral ligament with deep fascia around the lateral tibial plateau to avoid instability of the knee joint. We completely debrided bone splinters and necrotic soft tissue from the lower right limb and smoothed the edges. The free fibular head was fixated to the distal fibula with a fibular plate. The reconstructed inferior tibiofibular joint was reinforced with 3 lag screws before anastomosis of the vessels in the transplanted fibular head with the recipient area. Lastly, we made a suture flap in the wound and inserted rubber drainages (Fig. 3).

**Figure 4:**
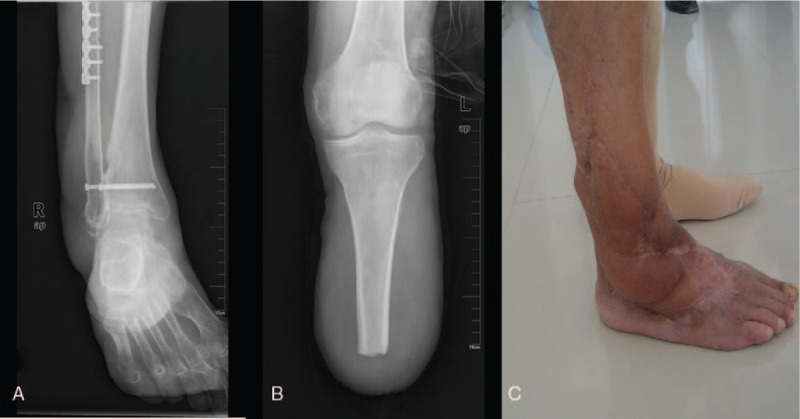
(A) One year later, internal fixation of bone fractions are stable and firm. New right ankle joint is satisfactory. (B) One year later, the left knee joint is satisfactory. (C) One year later, the patient can walk with the help of the prosthesis.

Due to necrosis of the skin and soft tissue of the distal flap 7 days after the operation, part of the reconstructed external ankle bone was exposed, so we transferred the sural neurocutaneous vascular flap to repair the skin and soft tissue defects of the lateral malleolus. X-ray examination 3 months after the operation showed that the fracture had a bony union and the shape of the transplanted fibular head was similar to the normal external ankle. At 12-month follow-up, walking function had been recovered with the help of a left artificial limb (Fig. 4). According to the Baird–Jackson scoring system, the curative effect was satisfied.

## 3 Discussion

Defects of the lateral malleolus are commonly caused by severe trauma and wide resections of neoplasms that destruct the integrity and stability of the ankle, thus the occurrence of traumatic osteoarthritis is inescapable and ankle function is seriously damaged.

A main principle of repairing defects of the lateral malleolus is rehabilitating the skeletal structure and mortise of the ankle. The fibular head is similar in shape to the lateral malleolus,^[6]^ and their largest circumference and transverse and vertical diameters are close in size. The difference between the shape of the lateral malleolus and the head of the fibula can gradually change because of joint movement in postoperative rehabilitation.^[7]^ The fibular head is not attached directly to the lateral meniscus, it is separated by the popliteus tendon. The fibular collateral ligament can be stitched to the superior tibiofibular joint capsule after excising the fibular head, which plays a relatively minor role in stabilizing the knee joint. Therefore, removing the fibular head has a minimal effect on knee joint function. In this case, we reconstructed the right lateral malleolus using the left fibular head. The patient had only the right ankle remaining, so reconstructing the ankle was very important to him because the flexibility of the right ankle joint could benefit the function of the left lower limb prosthesis. Fortunately, utilizing the contralateral fibular head to reconstruct the lateral malleolus was feasible. The superior segment of the fibula is well supplied with blood.^[8]^ The main blood vessels include the lateral inferior genicular artery and peroneal artery, which can both be applied to a fibular graft.^[9]^ The blood supply area of the peroneal artery is bigger than that of the lateral inferior genicular artery, which is important for a peroneal artery perforator flap. A skin flap of elliptical shape and a maximum size of 20 × 10 cm can be harvested along with the fibular graft from the lateral aspect of the leg.^[10]^ A peroneal artery perforator flap has the following advantages: the blood vessel diameter is thick enough for easy anastomosis; as long as the flap donor site is in good condition, the area is large enough to repair maxillofacial soft tissue defects; the flap color, texture, and elasticity are good; there is a minimal effect on the function and appearance of the donor area; blood can simultaneously be supplied to the flap and the fibular head, which helps to reduce infection; and there is a thin subcutaneous fatty layer, which is suitable for the external ankle with less skin and soft tissue.

In conclusion, we believe that using a combined composite flap of the vascularized fibular head to reconstruct the lateral malleolus is a good choice.
